# Supplementary material for: Molecular Doping of CVD-Graphene Surfaces by Perfluoroalkyl-Substituted Perylene Diimides Derivatives
Source: Nanomaterials (Basel). 2022 Nov 28;12(23):4239. doi: 10.3390/nano12234239 (PMC9737924; doi:10.3390/nano12234239)
Supplement: Supplementary file 1 [file nanomaterials-12-04239-s001.zip › nanomaterials-2050467-supplementary.pdf]

Molecular Doping of CVD-Graphene Surfaces by Perfluoroalkyl-Substituted Perylene Diimides Derivatives

Federico Chianese [1,2,*], Lucrezia Aversa [3], Roberto Verucchi [3,*] and Antonio Cassinese [1,2]

1 Dipartimento di Fisica, Università Degli Studi di Napoli Federico II, Piazzale Tecchio 80, 80125 Napoli, Italy
2 CNR-SPIN, Unità di Napoli, Piazzale Tecchio 80, 80125 Napoli, Italy
3 Institute of Materials for Electronics and Magnetism, CNR-IMEM, FBK Trento Unit, Via alla Cascata 56/C, 38123 Trento, Italy
* Correspondence: federico.chianese@unina.it (F.C.); roberto.verucchi@imem.cnr.it (R.V.)

Supplementary Materials

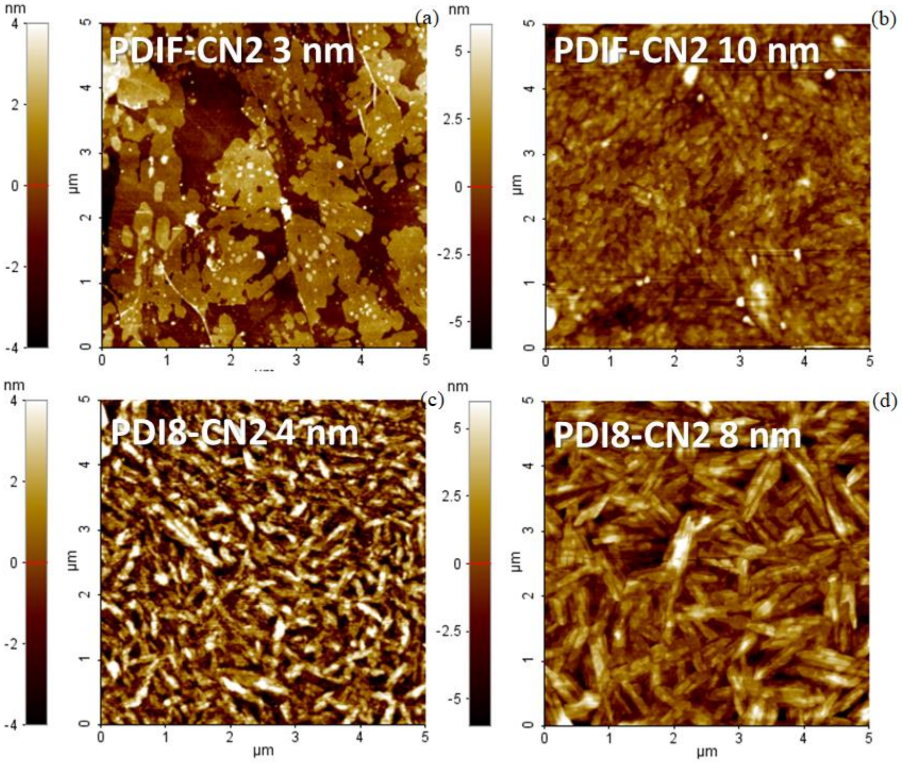

Figure S1. NC-AFM topography of PDI8-CN2(**c**–**d**) and PDIF-CN2 (**a**–**b**) grown on CVD-Graphene for different nominal thin film thicknesses.

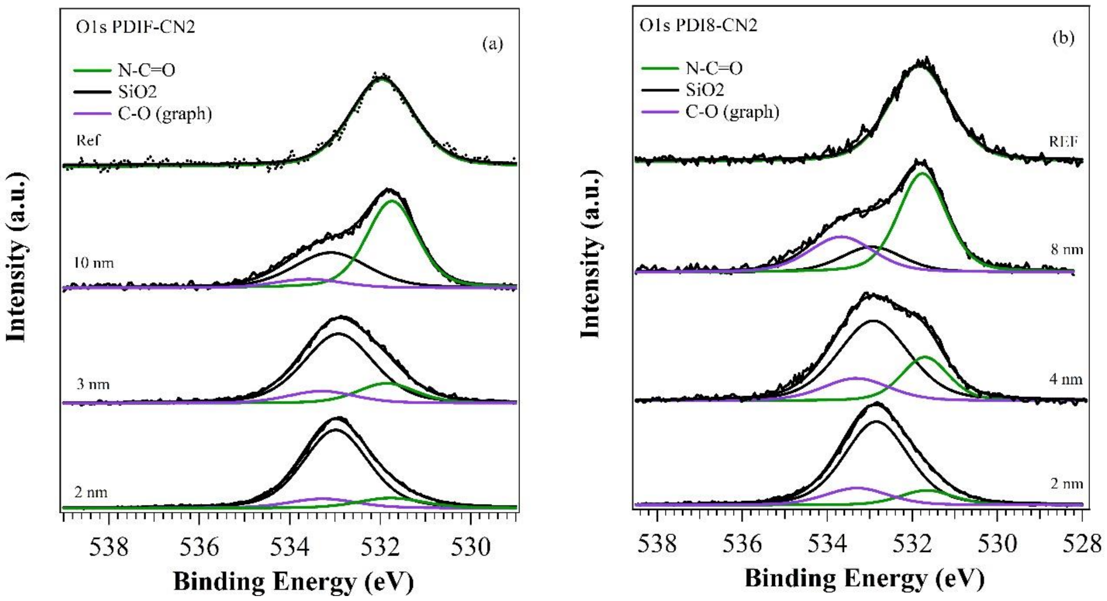

Figure S2. XPS O1s core levels spectra for (**a**) PDIF-CN2 and (**b**) PDI8-CN2 thin films grown on CVD-Graphene substrates.

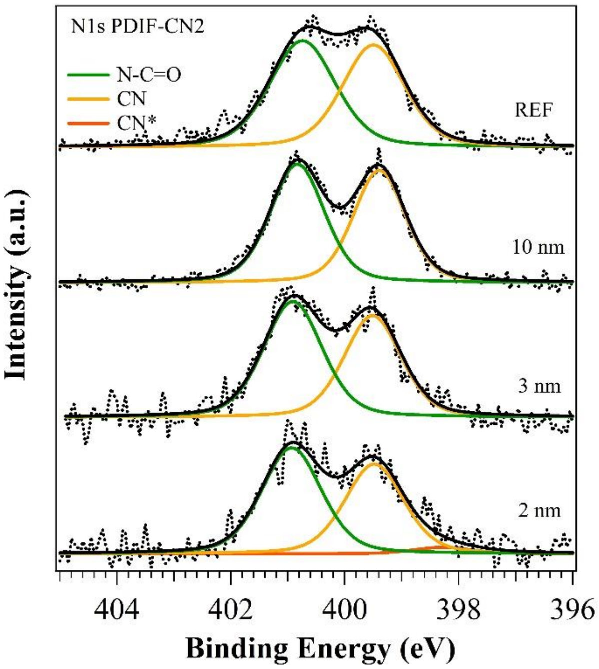

Figure S3. XPS N1s core level spectra for PDIF-CN2 thin film grown on CVD-Graphene substrates.

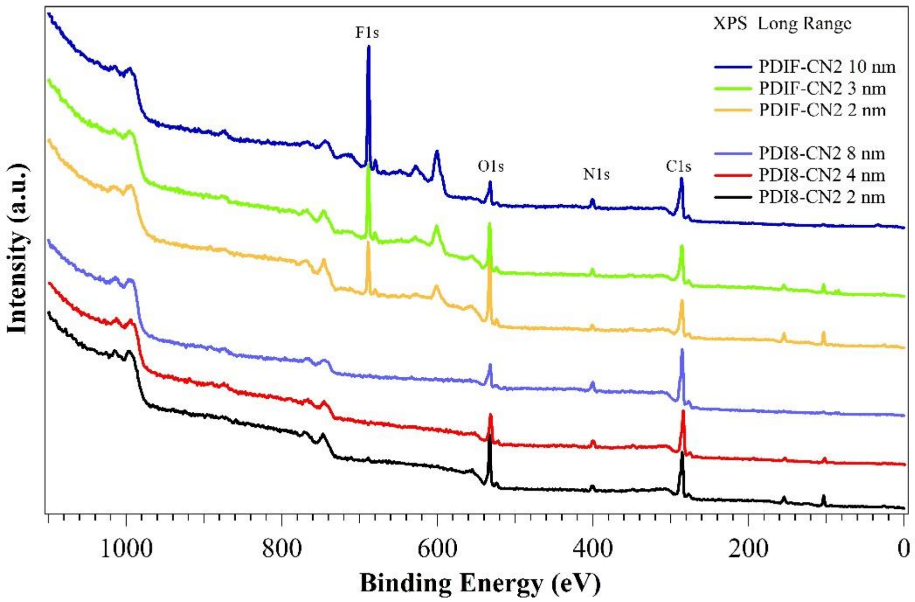

Figure S4. Long Range XPS spectra of PDI8-CN2 and PDIF-CN2 films grown on graphene substrate and at various thicknesses.

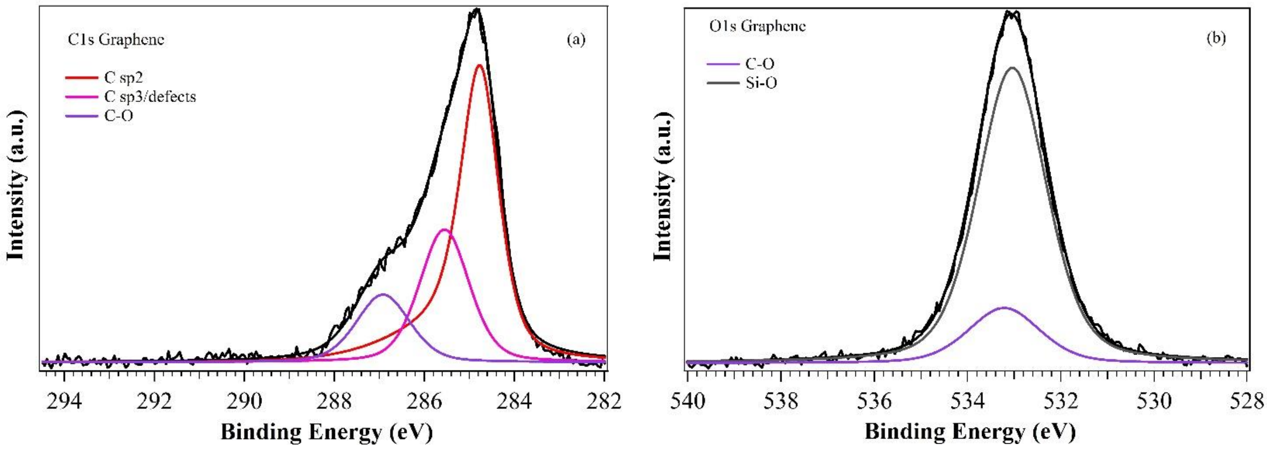

Figure S5. (**a**) C1s and (**b**) O1s core level spectra of the graphene substrate.

**PDIF-CN2**	**2nm**		**3nm**		**10nm**	
**C1s**	BE [eV]	FWHM [eV]	BE [ev]	FWHM [eV]	BE [eV]	FWHM [eV]
C-C	285.61	1.55	285.69	1.60	285.47	1.60
CON	288.75	1.50	288.69	1.55	288.56	1.50
CN	286.56	1.50	286.64	1.50	286.42	1.50
CF2	290.86	1.38	291.01	1.40	290.80	1.41
CF3	293.42	1.20	293.5	1.22	293.26	1.00
Graph C=C	284.70	1.00	284.65	1.01	284.78	1.06
Graph. sp^3	285.50	1.22	285.43	1.15	285.60	1.16
Graph. Ox.	286.85	1.48	286.80	1.47	286.93	1.40
**O1s**						
SiO_2	532.98	1.70	532.91	1.88	533.25	1.97
O in mol	531.76	1.71	531.91	1.55	531.74	1.32
Graph O	533.27	1.78	533.2	1.75	533.35	1.75
**N1s**						
CN*	398.28	1.22	-		-	
CN	399.48	1.22	399.51	1.19	399.39	1.1
N-CO	400.93	1.24	400.91	1.22	400.83	1.16
**F1s**						
CF_3	688.36	1.65	688.28	1.53	688.14	1.53
CF_2	689.06	1.85	689.09	1.67	688.89	1.70

Table S1. Details of core levels deconvolution (BE position and FWHM) for PDIF-CN2 films with thicknesses of 2 nm, 3 nm and 10 nm.

PDI8-CN2		2nm		4nm		8nm
**C1s**	BE [eV]	FWHM [eV]	BE [eV]	FWHM [eV]	BE [eV]	FWHM [eV]
C=C arom	284.36	1.00	284.42	1.00	284.34	1.00
C-C chain	285.74	1.30	285.80	1.25	285.60	1.30
C-H ring	284.82	1.00	284.88	1.05	284.75	1.10
CON	288.51	1.35	288.57	1.45	288.53	1.35
CN	286.76	1.25	286.82	1.30	286.78	1.25
Other C	286.31	1.20	286.37	1.35	286.33	1.25
Graph C=C	284.79	1.10	284.72	1.05	284.92	1.10
Graph. sp3	285.57	1.23	285.46	1.16	285.7	1.23
Graph ox.	286.94	1.45	286.87	1.45	287.07	1.40
**O1s**						
SiO_2	532.84	1.78	533.11	2.20	533.34	2.06
O in mol	531.66	1.50	531.8	1.30	531.76	1.41
Graph O	533.30	1.75	533.10	2.00	533.47	1.83
**N1s**						
CN*	398.25	1.20	-	-	-	-
CN	399.43	1.15	399.34	1.17	399.42	1.21
N-CO	400.70	1.14	400.64	1.15	400.69	1.20

Table S2. Details of core levels deconvolution (BE position and FWHM) for PDI8-CN2 films with thicknesses of 2 nm, 4 nm and 10 nm
